# scGlycoAI: machine learning analysis of single-cell glycan and RNA sequencing data for cell-type annotation and malignancy-associated glycan discovery

**DOI:** 10.1093/glycob/cwag100

**Published:** 2026-10-24

**Authors:** Dinh Xuan Tuan Anh, Hiroaki Tateno

**Affiliations:** Cellular and Molecular Biotechnology Research Institute, National Institute of Advanced Industrial Science and Technology (AIST), Central 6, 1-1-1 Higashi, Tsukuba, Ibaraki 305-8566, Japan; PhD Program in Human Biology, School of Integrative and Global Majors, University of Tsukuba, 1-1-1 Tennodai, Tsukuba, Ibaraki 305-8575, Japan; Cellular and Molecular Biotechnology Research Institute, National Institute of Advanced Industrial Science and Technology (AIST), Central 6, 1-1-1 Higashi, Tsukuba, Ibaraki 305-8566, Japan; Cooperative Graduate School Program, University of Tsukuba, 1-1-1 Tennodai, Tsukuba, Ibaraki 305-8575, Japan

**Keywords:** cell-type annotation, machine learning, pancreatic ductal adenocarcinoma, scGR-seq, single-cell glycomics

## Abstract

Single-cell glycan and RNA sequencing (scGR-seq) enables characterization of cellular glycan heterogeneity. Here, we present scGlycoAI, an interpretable machine learning framework that integrates scGR-seq data for glycan-centered biological discovery. Using lectin-binding profiles as input, scGlycoAI employs hierarchical machine learning to accurately classify cell types and subtypes within complex tumor microenvironments. Applied to pancreatic ductal adenocarcinoma (PDAC) datasets, scGlycoAI achieved an overall eight-class classification accuracy of 90.3% under leave-one-patient-out validation using glycan features alone. These results demonstrate that glycan signatures, despite their lower dimensionality compared with transcriptomic profiles, are sufficient for robust cell-type annotation and represent cell-type-specific molecular signatures. Feature importance analysis identified PVL lectin binding, which reflects recognition of sialylated and/or GlcNAc-containing glycans, as a key feature of aggressive cancer states. A neural-network model combined with SHAP-based interpretation further revealed that elevated PVL binding was associated with transcriptional programs linked to epithelial–mesenchymal transition, KRAS signaling, extracellular matrix remodeling, and inflammatory responses. Higher expression of the PVL-associated gene signature was associated with poorer overall survival in the TCGA pancreatic adenocarcinoma cohort. Collectively, these findings suggest that glycan-based machine learning enables accurate cell classification while providing biological insights into malignancy-associated glycan states at single-cell resolution. This framework provides a foundation for glycan-centered biomarker discovery and glycan-targeted therapeutic development.

## Introduction

Glycans represent a critical yet underexplored dimension of cellular heterogeneity. As highly diverse and dynamic cell-surface molecules, they regulate essential biological processes, including cell–cell communication, receptor signaling, and immune recognition (Varki et al. 2022; Wu et al. 2026). In cancer, aberrant glycosylation is a hallmark of disease progression and contributes to invasion, metastasis, and immune evasion (Munkley and Elliott 2016; Qusairy and Rada 2025; Rodriguez 2025). Previous studies using lectin-based profiling and mass spectrometry have demonstrated that distinct cell populations exhibit characteristic glycan signatures, suggesting that glycans represent a cell-type-informative molecular layer that complements transcriptomic identity (Keisham et al. 2024; Sojitra et al. 2024; Anh et al. 2026).

Recent advances in single-cell glycan and RNA sequencing (scGR-seq) enable simultaneous measurement of transcriptomic and glycan features, offering unprecedented opportunities to investigate glycan-associated cellular diversity at single-cell resolution (Minoshima et al. 2021; Odaka et al. 2022; Keisham et al. 2024; Odaka and Tateno 2025; Anh et al. 2026; Anh and Tateno 2026). However, systematic frameworks to leverage such multimodal data for cell-type annotation and glycan-centered biological discovery remain limited.

In parallel, machine learning (ML) approaches have emerged as powerful tools for integrating high-dimensional biological data and uncovering complex molecular relationships. Importantly, interpretable frameworks such as SHAP (SHapley Additive exPlanations) enable identification of features that drive model predictions, providing mechanistic insight beyond conventional analyses (Lundberg et al. 2020). While these approaches have been widely applied to transcriptomic datasets, their application to single-cell glycomic data and glycan-defined cellular states remains largely unexplored.

Previously, Qin et al. applied a deep learning model to single-cell RNA sequencing data integrated with SUGAR-seq–derived DNA-barcoded PHA-L lectin measurements, thereby elucidating the structural features and biological roles of branched N-glycans (Qin et al. 2022). Building on this concept, we present scGlycoAI, an interpretable AI framework that integrates lectin-binding profiles from 29 lectins with RNA sequencing data at single-cell resolution obtained via scGR-seq to enable glycan-based cell-type annotation and biological discovery. By leveraging lectin-binding profiles and hierarchical machine learning, scGlycoAI accurately classifies cell types and subtypes within complex tumor microenvironments. Applying this framework to scGR-seq datasets from patients with pancreatic ductal adenocarcinoma (PDAC), we further identify PVL-associated glycans as key determinants of aggressive cancer cell states. Integrative neural network–based machine learning and SHAP analyses reveal transcriptional programs associated with these glycan states, linking glycosylation to epithelial–mesenchymal transition, oncogenic signaling, and poor patient prognosis.

Collectively, this study establishes a glycan-centered AI framework for improving cell annotation and uncovering malignancy-associated glycan programs at single-cell resolution, providing a foundation for glycan-based biomarker discovery and therapeutic targeting.

## Results

### Glycans enable hierarchical cell annotation and predictive modeling

To systematically investigate the functional roles of glycans in cancer and identify clinically relevant glycan targets, we developed scGlycoAI, an AI-driven single-cell glycomic–transcriptomic framework that integrates machine learning–based cell annotation with neural-network model–guided glycan discovery. By combining single-cell glycan profiling with transcriptomic analysis, scGlycoAI enables comprehensive characterization of cancer-associated glycans within complex tumor microenvironments (TMEs).

The scGlycoAI framework comprises five analytical steps (Fig. 1A). First, scGR-seq data are generated to capture paired transcriptomic and glycomic features. Second, lectin-binding intensities are quantified to define cell-surface glycan signatures. Third, these signatures are leveraged for machine learning–based annotation of cell types and cellular states. Fourth, neural-network models link glycan-defined cellular states to malignant transcriptional programs and prioritize candidate genes or pathways for further investigation. Finally, SHAP (SHapley Additive exPlanations) analysis enables interpretation of model outputs, revealing key glycan features and their associated biological pathways. Together, this framework provides an interpretable platform linking glycan landscapes to cancer biology at single-cell resolution.

**Figure 1:**
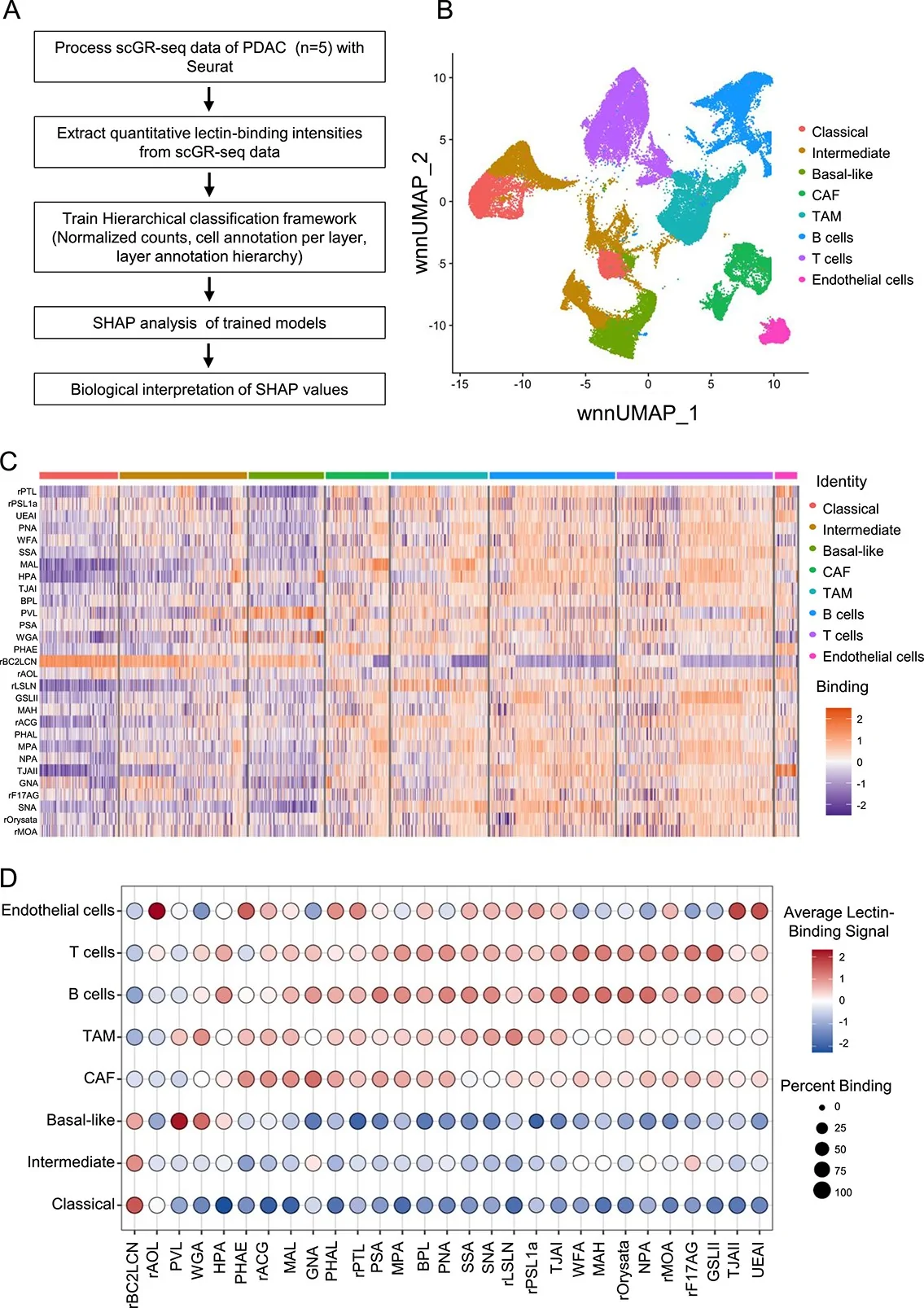
Distinct glycan landscapes and lectin-binding signatures in PDAC. (A) Schematic overview of the scGlycoAI workflow. (B) UMAP visualization of integrated single-cell glycomic and transcriptomic data from five PDAC patients. (C) Heatmap showing lectin binding intensities across all cell types within the TME. Lectins are listed on the *y*-axis. The gradient indicates binding intensity, from high to low. (D) Dot plot showing lectin binding intensities across annotated cell types. Dot size represents the proportion of cells with detectable lectin binding above the lectin-specific background threshold, whereas the gradient represents the average normalized lectin-binding signal. Accordingly, a high fraction of lectin-positive cells does not necessarily indicate strong lectin binding.

To evaluate the utility of glycan features for cell-type annotation, we analyzed a scGR-seq dataset from patients with pancreatic ductal adenocarcinoma (PDAC) (*n* = 5) (Fig. 1B) (Anh et al. 2026). The present study represents a secondary computational analysis of this previously reported dataset and focuses on the development and evaluation of the scGlycoAI framework rather than the generation of new experimental data (Anh et al. 2026). The original study annotated the dataset into 19 cell types primarily based on transcriptomic profiles. In the present analysis, we focused specifically on the ability of lectin-binding features to distinguish cellular populations. Following quality control and normalization, we analyzed the glycomic profiles independently of the transcriptomic annotation. Because the lectin panel provides a smaller and broader feature space than transcriptomic profiling, closely related cell types exhibited overlapping glycan signatures and could not be reliably resolved at the same resolution as the 19 transcriptome-defined populations. Importantly, the remaining transcriptome-defined cell types were not excluded from the analysis but were consolidated into broader categories because closely related populations exhibited overlapping glycan signatures and could not be robustly distinguished using lectin-binding features alone. We therefore consolidated these populations into eight major cell-type categories representing the broader immune, stromal, and cancer compartments. This glycome-based classification was used to assess the discriminatory capacity of lectin-binding features rather than to redefine the original transcriptomic cell-type annotation. The integrated visualization was retained because transcriptome-derived annotations provide the reference cellular identities against which glycan-based features can be trained and evaluated, enabling assessment of the predictive power of lectin-binding profiles. The relative abundance of these major populations varied across patients, consistent with the pronounced interpatient heterogeneity characteristic of PDAC (Anh et al. 2026).

Lectin-binding analyses revealed pronounced differences in glycan signatures across cell populations (Figs. 1C, D). Because dot size and color intensity represent different measurements, a high proportion of lectin-positive cells does not necessarily indicate strong lectin binding. Distinct patterns were observed not only between malignant and nonmalignant cells but also among stromal and immune populations, cancer subtypes, and even within individual cell types. Cancer cells exhibited relatively restricted lectin-binding profiles, whereas nonmalignant populations showed broader and more diverse lectin reactivity, consistent with the aberrant and selective glycosylation landscape of malignancy.

Importantly, this heterogeneity in lectin-binding profiles provides an additional layer of biological resolution beyond transcriptomic data alone. These findings demonstrate that scGR-seq–derived glycan signatures serve as informative features for accurate cell-type annotation and stratification of cellular states in PDAC.

### scGlycoAI accurately predicts cell identities across hierarchical annotation layers

To determine whether lectin-binding profiles alone can enable accurate cell-type annotation, we applied the scGlycoAI framework to scGR-seq datasets. To minimize information leakage, model performance was primarily evaluated using leave-one-patient-out validation, in which four patients were used for training and one independent patient was used for testing. Hierarchical classifiers based on XGBoost were sequentially trained across multiple annotation layers (Figs. 2A, B).

**Figure 2:**
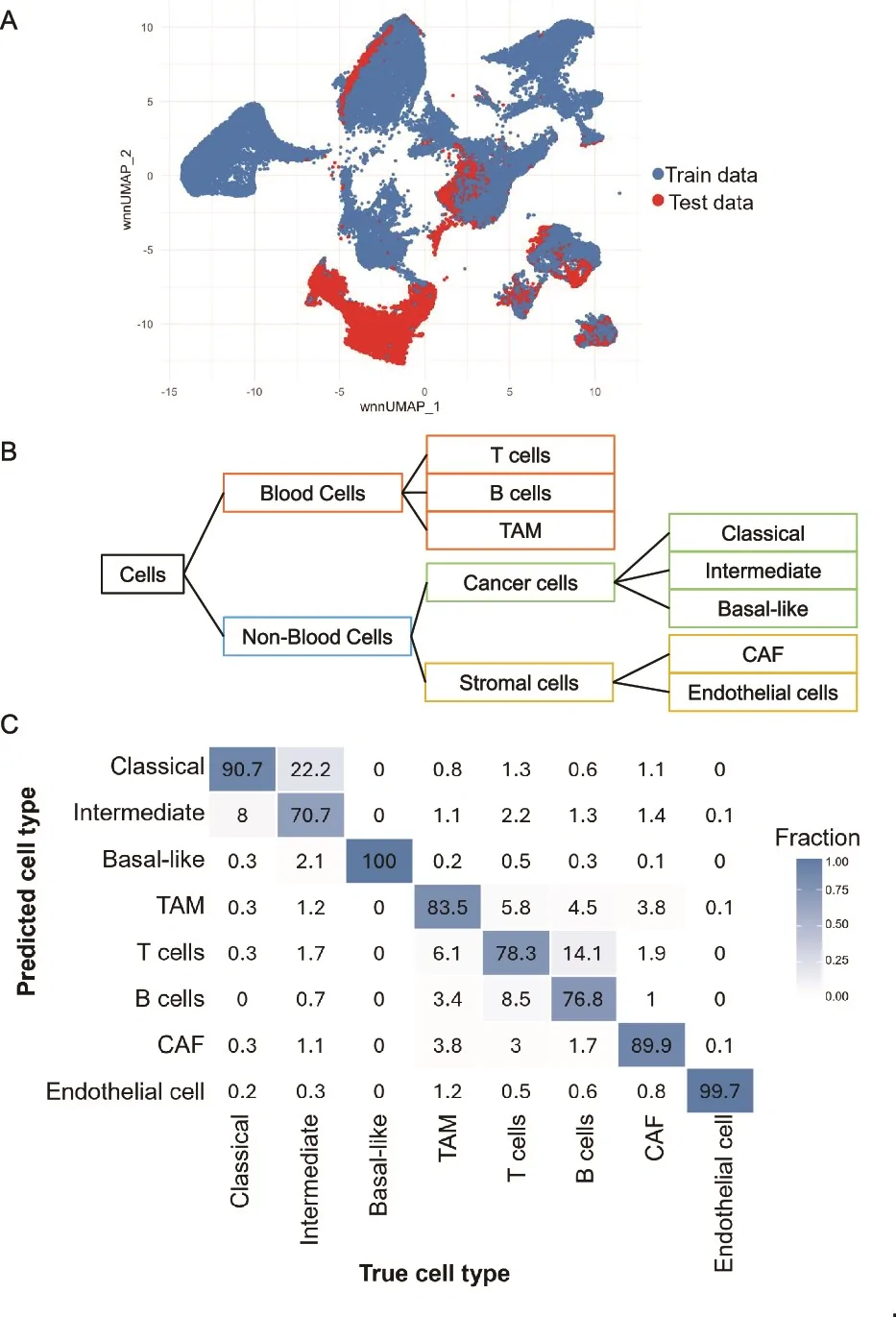
Hierarchical cell-type prediction using lectin-binding profiles. (A) UMAP visualization showing the training-patient and held-out test-patient cells used in patient-level validation. (B) Schematic of the leave-one-patient-out training and testing workflow and the hierarchical classification structure. Parent nodes represent broad cellular compartments, and terminal nodes represent the eight final cell classes. (C) Confusion matrix showing the performance of the final eight-class classifier on held-out patients in the leave-one-patient-out validation scheme after integration of all hierarchical prediction layers.

Cells were first classified into broad cellular compartments and subsequently assigned to finer cell populations within the corresponding compartment. This hierarchical design reduced classification complexity at each level and enabled the model to focus on distinguishing biologically related populations based on their lectin-binding profiles.

For prediction, scGlycoAI uses normalized lectin-binding profiles to perform stepwise classification across annotation layers. At each step, predictions are assigned based on maximum probability, and a rejection threshold (0.5) is applied to exclude low-confidence assignments, which are labeled as “unclassified.” Even under the stringent leave-one-patient-out validation scheme, scGlycoAI achieved high classification performance, with an overall eight-class accuracy of 90.3% under leave-one-patient-out validation. Consistently high AUC values from one-versus-rest ROC analyses further demonstrated the strong discriminative capability of lectin-based features for cell-type annotation (Fig. 2C, Supplementary Fig. 1). Importantly, classification performance was not driven solely by the separation of highly distinct cell populations. Closely related tumor-cell subtypes, including classical, intermediate, and basal-like cancer cells, were also distinguished with high discriminative performance, with one-versus-rest AUC values of 0.824, 0.854, and 0.999, respectively (Supplementary Fig. 1). These results indicate that scGlycoAI can resolve biologically similar cellular states in addition to broad cellular compartments. The final cell-type prediction was obtained by integrating the predictions across the hierarchical levels, resulting in eight final cell-type categories. Cells that did not meet the predefined classification criteria were designated as unclassified/rejected and were not counted as correctly classified cells. Overall, 7.8% (3998 of 51,265 cells) of cells were categorized as unclassified. These results demonstrate that lectin-binding signatures alone contain sufficient biological information and capture cell-type-associated glycan features that robustly distinguish diverse cellular populations. This finding further suggests that glycan profiles are inherently cell-type-specific and encode discriminatory features that define cellular identity. Interestingly, the model achieved approximately 90% accuracy when trained and evaluated using a stratified 80:20 cell-level training–test split, further supporting the ability of lectin-binding signals to provide biologically informative features for accurate cell-type annotation (Supplementary Fig. 2).

To identify the glycan features driving these predictions, we performed feature importance analysis. PVL lectin binding, which recognizes both sialic acid- and GlcNAc-containing glycan structures, emerged as a key feature for distinguishing basal-like and intermediate PDAC subtypes associated with aggressive tumor phenotypes (Supplementary Fig. 3) (Guerrero et al. 2020; Ruta et al. 2024; Felipe and Kalisz 2026). PVL binding was consistently higher in basal-like pancreatic cancer cells compared with classical and intermediate cancer cells across all patient samples (Supplementary Fig. 4). This finding is consistent with previous observations that PVL-binding glycans are enriched in basal-like PDAC (Anh et al. 2026).

Together, these results highlight the importance of PVL-reactive glycan structures in defining aggressive cancer cell states and provide a rationale for further investigation of their potential roles in PDAC progression.

### scGlycoAI reveals the biological significance of PVL-associated glycan states

To investigate the transcriptional programs associated with PVL lectin binding in PDAC cells, we established a machine learning–based classification framework using single-cell gene expression profiles. Cancer cells were stratified into PVL-high and PVL-low populations according to PVL lectin-binding intensity, with the top and bottom 20% of cells defined as PVL-high and PVL-low, respectively (Fig. 3A). This strategy enabled focused characterization of the molecular features associated with elevated PVL binding at single-cell resolution.

**Figure 3:**
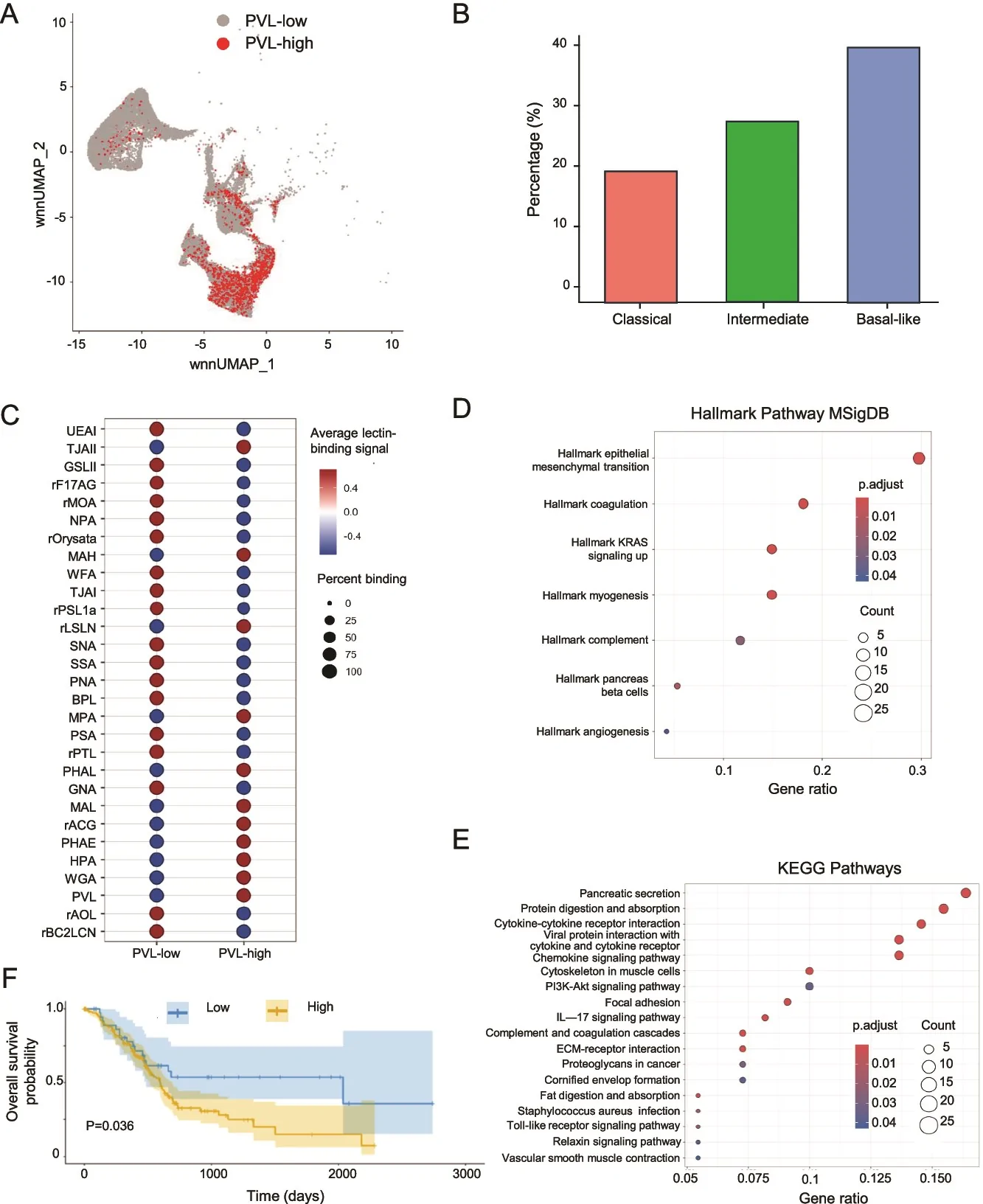
SHAP-based interpretation reveals biological mechanisms underlying PVL-associated glycan signatures. (A) UMAP visualization of PVL lectin-binding groups within the cancer cell population. (B) Bar plot showing PVL-binding intensity across different cancer cell subtypes. (C) Dot plot showing lectin-binding patterns between PVL-low and PVL-high cancer cell groups. Dot size represents the proportion of cells with detectable lectin binding, while color intensity indicates the average CLR-normalized and scaled lectin-binding signal. The color-scale values represent relative normalized lectin-binding intensities and should not be interpreted as fold changes. (D–E) Hallmark and KEGG pathway enrichment analyses of the top 200 SHAP-prioritized genes identified from the PVL-high group. (F) Kaplan–Meier overall survival analysis of pancreatic adenocarcinoma (PAAD) patients stratified according to the expression levels of the 200 SHAP-prioritized genes associated with the PVL-high state.

Analysis of PVL-binding intensity across PDAC cancer subtypes revealed a progressive increase from classical to intermediate and basal-like cancer cells, with basal-like cells showing the highest PVL signal (Fig. 3B). These findings support the enrichment of PVL-recognized glycan structures in aggressive cancer states. Because PVL can recognize sialylated and GlcNAc–containing glycans, elevated PVL binding is consistent with alterations in one or both of these glycan features in basal-like PDAC cells. However, lectin-binding measurements alone do not determine their relative contributions.

To further characterize the glycomic features associated with PVL-high cells, we compared lectin-binding profiles and glycosyltransferase expression between PVL-high and PVL-low populations (Fig. 3C and Supplementary Fig. 5). Multiple glycosyltransferase genes were differentially expressed between the two populations. Among sialyltransferases, *ST3GAL4* expression was significantly elevated in PVL-high cells. Given the reported preference of PVL for α2,3-sialylated glycans (Anh et al. 2026), this observation suggests that increased PVL binding is associated with enhanced α2,3-sialylation.

In addition, several galactosyltransferase genes, including *B3GALT5*, *B4GALT1*, *B4GALT4*, and *B4GALT5*, were downregulated in PVL-high cells, whereas GlcNAc-related glycosyltransferase genes, including *MGAT1*, *MGAT2*, *MGAT4B*, and *B3GNT5*, were upregulated. This expression pattern is consistent with increased exposure of GlcNAc-containing glycans and may contribute to enhanced binding of PVL.

Consistent with these transcriptomic changes, lectins recognizing α2,3-linked sialic acid (MAL, MAH, and rACG) as well as the GlcNAc-binding lectin (WGA) showed increased binding in PVL-high cells (Fig. 3C). Furthermore, PVL binding was positively correlated with the binding of rACG, MAL, MAH, and WGA across tumor subtypes and individual patients, although the strength of these correlations varied considerably among subtypes and individuals (Supplementary Figs. 6 and 7). Collectively, these results indicate that PVL binding reflects a distinct glycomic state characterized by elevated α2,3-sialylation and altered GlcNAc-associated glycan structures. This glycomic signature is enriched in aggressive PDAC cell populations and may contribute to the biological characteristics associated with tumor progression.

We next trained a multilayer perceptron neural network to classify cancer cells according to PVL-binding status using single-cell transcriptomic profiles. The dataset was divided into training and test sets, and the model learned transcriptional patterns that distinguished PVL-high from PVL-low cells. Robust classification performance indicated that PVL-associated glycan states are closely coupled to distinct transcriptional programs in PDAC cells.

To interpret the biological features underlying model predictions, we performed SHAP analysis on the trained neural network. SHAP values quantified the contribution of individual genes to PVL-high classification, allowing identification of genes most strongly associated with elevated PVL binding. Pathway enrichment analysis of the top 200 SHAP-prioritized genes revealed strong enrichment of programs associated with aggressive tumor phenotypes, including epithelial plasticity, inflammatory signaling, extracellular matrix remodeling, and glycan-associated malignant processes (Supplementary Table 1).

Hallmark pathway analysis showed that epithelial–mesenchymal transition (EMT) was the most significantly enriched program in PVL-high cells (Fig. 3D). EMT is strongly associated with invasion, metastasis, cellular plasticity, and therapeutic resistance in PDAC (Palamaris et al. 2021; Yang et al. 2023). In addition, enrichment of KRAS signaling further supported the malignant nature of PVL-high cells, as oncogenic KRAS activity is a central driver of PDAC progression. Other enriched pathways, including coagulation, complement, angiogenesis, and myogenesis, suggested activation of TME remodeling and stromal interaction programs.

Consistent with these findings, KEGG pathway analysis revealed enrichment of cytokine signaling, chemokine signaling, focal adhesion, ECM–receptor interaction, PI3K–Akt signaling, and proteoglycans in cancer (Fig. 3E). These pathways are involved in cancer cell survival, migration, invasion, inflammatory communication, and interactions with the extracellular matrix. Together, these results suggest that PVL-high cells exhibit transcriptional programs associated with invasive behavior, inflammatory signaling, and tumor-promoting glycan remodeling.

We further assessed the clinical relevance of the PVL-associated transcriptional program using the TCGA pancreatic adenocarcinoma cohort. Patients with lower expression of the 200 SHAP-prioritized genes showed significantly improved overall survival compared with patients with higher expression levels (Fig. 3F). These findings indicate that genes associated with PVL-high glycan states are not only linked to single-cell glycan phenotypes but also correlate with poor clinical outcomes in PDAC.

To assess the robustness of the PVL threshold and program analysis, the top 10% of PVL-binding cells were defined as PVL-high and the bottom 10% as PVL-low, with four patients used for training and one independent patient for testing. The resulting gene program was consistent with the SHAP-identified genes and showed enrichment in cancer progression-related pathways in both MSigDB Hallmark and KEGG analyses. Furthermore, patients with lower expression of the SHAP gene signature showed better survival than those with higher expression (Supplementary Fig. 8).

To further investigate the biological relevance of PVL within a defined tumor-cell population, we repeated the analysis using only classical PDAC cells, thereby minimizing potential confounding effects arising from differences in cellular composition. Notably, PVL-high classical cells exhibited enrichment of gene programs and pathways associated with cancer progression (Supplementary Fig. 9). Consistent with these findings, patients with higher expression of the PVL-associated SHAP gene signature showed poorer survival compared with those with lower expression (Supplementary Fig. 9). Together, these results further support an association between the PVL-associated molecular program and aggressive pancreatic cancer biology.

Functional characterization of the top 50 genes among the 200 SHAP-prioritized genes further supported the association between PVL binding and aggressive tumor biology (Supplementary Table 1). Spearman correlation analysis, treating PVL-binding intensity as a continuous variable, revealed significant positive correlations between PVL-binding intensity and the expression of multiple SHAP-prioritized genes (Supplementary Fig. 10, Supplementary Table 2), further supporting the biological relevance of the identified PVL-associated transcriptional program. Several highly ranked genes, including *KRT17*, *KRT16*, and *S100A2*, are established markers of basal-like or squamous-like PDAC states associated with poor prognosis (Chen et al. 2023; Delgado-Coka et al. 2024; Romashin et al. 2024). In addition, mucin-associated genes such as *MUC5AC*, *MUC6*, *TFF1*, *TFF2*, and *TFF3* were prominent among the predictive features, consistent with altered glycoprotein and sialylation programs in aggressive gastrointestinal cancers (Arumugam et al. 2011; Rachagani et al. 2012; Jahan et al. 2019). Genes involved in extracellular matrix remodeling and invasion, including *MMP1*, *MMP12*, *HAS1*, *SPARC*, and *COL1A2*, were also enriched (Vaz et al. 2015; Sato et al. 2016; Xie et al. 2021; Luan et al. 2023; Li et al. 2025), along with inflammatory and immune-related genes such as *CXCL9*, *CXCL10*, *CXCL14*, *CCL4*, and *S100A9* (Mukaida et al. 2020; Jing et al. 2022; Kung et al. 2022).

Collectively, these findings demonstrate that SHAP-guided interpretation of machine learning models captures both the glycan landscape and malignant transcriptional networks associated with PVL-binding glycan–enriched PDAC cell states. Thus, PVL-associated glycan signatures mark aggressive cancer cell populations characterized by EMT, inflammatory signaling, extracellular matrix remodeling, and poor patient prognosis.

## Discussion

In this study, we present scGlycoAI, a computational framework that enables cell-type annotation based on glycomic profiles derived from single-cell glycan–RNA sequencing (scGR-seq) data. In contrast to conventional approaches that rely predominantly on transcriptomic information, scGlycoAI leverages lectin-binding signatures to classify cells across hierarchical annotation layers. Because hierarchical cellular organization is broadly conserved across tissues and biological contexts, this framework provides a flexible and scalable strategy for glycan-based cell annotation. The hierarchical approach achieved higher classification accuracy than the conventional multiclass model, with overall accuracy of 90.3% and 80.4%, respectively, under leave-one-patient-out validation (Supplementary Fig. 11).

Our findings establish that glycan profiles represent cell-type-specific molecular signatures capable of defining cellular identity at single-cell resolution. Importantly, this does not imply redundancy with transcriptomic information; rather, glycan profiles capture a complementary and partially independent layer of cellular information that reflects integrated regulatory processes beyond gene expression alone and is closely associated with the regulation of intercellular communication.

The ability of glycan features to support accurate classification likely arises from the coordinated regulation of glycosylation pathways, through which glycan structures encode the integrated output of transcriptional, post-transcriptional, and enzymatic processes. As such, glycan landscapes may serve as compact yet information-rich readouts of cellular states, enabling robust discrimination of cell identities despite the relatively limited dimensionality of glycomic features.

A key advantage of scGlycoAI is its capacity to achieve high classification performance using a comparatively small number of glycan features. In contrast to transcriptome-based approaches that require high-dimensional datasets, glycan-based models operate on lower-dimensional lectin-binding profiles while retaining biologically meaningful signals. These properties may be particularly advantageous in contexts where transcriptomic data are limited in quality or availability, highlighting the potential applicability of glycan-based approaches in clinical and translational settings. In particular, the cell-type-specific lectin-binding profiles identified in this study may be directly translatable to practical cell identification strategies using established platforms such as flow cytometry. The integration of lectin-based probes with fluorescence detection could enable glycan-guided cell classification in routine experimental and clinical workflows. Furthermore, the inherently multiplexed nature of lectin-binding measurements enables the simultaneous interrogation of diverse glycan structures, supporting high-resolution and combinatorial cell identification beyond conventional marker-based approaches. Nevertheless, this study is based on a limited number of samples, and dataset heterogeneity may influence model robustness. Further validation in larger cohorts and across diverse tissue types will be important to establish the generalizability of this framework. The difference in cell-type resolution reflects the complementary information captured by transcriptomic and glycomic modalities. Beyond cell-type annotation, scGlycoAI enables functional interrogation of disease-associated glycan states. In PDAC, we identified enrichment of PVL lectin binding, which reflects recognition of sialylated and/or GlcNAc-containing glycan structures, in basal-like cancer cells associated with aggressive tumor behavior. PVL-positive cells exhibited transcriptional programs linked to epithelial–mesenchymal transition (EMT), KRAS signaling, inflammatory responses, and extracellular matrix remodeling, indicating that PVL-binding glycan states are closely associated with malignant progression.

Integration of a neural-network model with SHAP-based interpretation further revealed transcriptional networks underlying PVL-associated glycan states. The SHAP-derived gene signatures captured both glycan-related features and malignant transcriptional programs enriched in PVL-binding glycan–high cancer cells. Notably, several predictive genes—including basal-like markers (such as *KRT17*, *KRT16*, and *S100A2*), mucin-associated genes (including *MUC5AC*, *MUC6*, and the TFF family), and extracellular matrix regulators (such as *MMP1*, *MMP12*, *SPARC*, and *COL1A2*)—have been implicated in aggressive PDAC phenotypes and glycan remodeling (Rachagani et al. 2012; Vaz et al. 2015; Sato et al. 2016; Jahan et al. 2019; Xie et al. 2021; Chen et al. 2023; Luan et al. 2023; Delgado-Coka et al. 2024; Romashin et al. 2024). These findings suggest that PVL-binding glycan states are associated with transcriptional programs related to tumor progression, invasion, and cellular plasticity. However, functional perturbation studies will be required to determine whether these glycan changes directly contribute to malignant phenotypes.

Furthermore, the enrichment of inflammatory and immune-related pathways indicates that PVL-associated glycan states may contribute to the regulation of tumor–microenvironment interactions. The association between SHAP-prioritized gene expression and poor patient prognosis further underscores the clinical relevance of these glycan-linked transcriptional programs. While comparison with simpler classifiers, such as logistic regression, may provide complementary information regarding predictive performance, the primary aim of the present study was to uncover biologically relevant genes associated with PVL-binding status rather than to identify the optimal predictive algorithm. Accordingly, we employed a simple neural-network framework coupled with SHAP analysis, which enabled simultaneous prediction of PVL-binding status and quantitative interpretation of gene-level contributions to the model. This interpretability-oriented strategy facilitated prioritization of genes potentially underlying PVL-associated transcriptional programs. Nevertheless, systematic benchmarking against logistic regression and other machine-learning approaches may provide additional insight into model selection and performance and therefore represent an important direction for future investigation. Importantly, the PVL-associated transcriptional program remained detectable even when the analysis was restricted exclusively to classical cancer cells. These findings support an association between PVL-reactive glycan features and pancreatic cancer progression that is not simply a surrogate for basal-like identity.

Although this study focuses on PDAC, the conceptual framework established here is likely broadly applicable across tissues and disease contexts. Given that glycosylation is a fundamental and ubiquitous feature of cell biology, the identification of cell-type-specific glycan signatures may represent a generalizable principle for defining cellular identity.

Collectively, our findings demonstrate that single-cell glycomics–based machine learning not only enables accurate cell-type annotation but also provides mechanistic insights into the relationship between glycosylation and malignant phenotypes. scGlycoAI thus offers a versatile platform for integrating glycomics with artificial intelligence, opening new avenues to dissect cellular heterogeneity and identify glycan-associated disease programs and therapeutic targets. Notably, the principal findings remained reproducible under leave-one-patient-out validation and after restricting the analysis exclusively to classical cancer cells, supporting the robustness of the identified PVL-associated transcriptional program.

## Materials and methods

### Seurat analysis for scGR-seq data

For scRNA-seq analysis, low-quality cells were removed to minimize contamination from dead cells. Specifically, cells expressing fewer than 200 genes and those with over 10% mitochondrial gene content were excluded from downstream analyses. The data were normalized using the “LogNormalize” method in the NormalizeData() function with a scale factor of 10,000. Following quality control, the top 3000 highly variable genes were identified using the FindVariableFeatures() function. The data were then scaled using ScaleData(). Dimensionality reduction was performed using principal component analysis (PCA), and the number of components used for downstream analysis was determined based on the elbow plot. A k-nearest neighbor (KNN) graph was constructed using the FindNeighbors() function on the first 8 principal components, followed by clustering with the FindClusters() function at a resolution of 1.0. The resulting clusters were visualized using UMAP.

For scGlycan-seq analysis, cells were filtered based on lectin-derived UMI features, retaining cells with 10–31 detected lectin features per cell, where a feature was considered detected when its raw UMI count was greater than zero. The mouse IgG_1_ negative-control measurement was retained during quality-control and background assessment and was subsequently excluded from downstream lectin feature analysis. The IgG_1_ control was used to characterize nonspecific/background signal and establish the detection threshold for defining lectin-positive cells. For each lectin, the detection threshold was defined using the corresponding mouse IgG1 negative-control signal (B). Cells were classified as lectin binders when the lectin-binding signal exceeded the background threshold (Xij > B), whereas cells with signals at or below the threshold were classified as non-binders (Xij ≤ B), where Xij denotes the signal intensity of lectin i in cell j and B represents the mouse IgG1 negative-control signal measured in the same cell. The data were then centered log-ratio (CLR) normalized using the NormalizeData() function and subsequently scaled using ScaleData(). Unlike scRNA-seq, the FindVariableFeatures() function was not applied due to the limited number of lectins (29 conventional lectins). PCA was used for dimensionality reduction, and the number of components was selected based on the elbow plot. A KNN graph was constructed using the FindNeighbors() function on the first 10 principal components, followed by clustering with the FindClusters() function at a resolution of 1.0. UMAP was then used for visualization.

For the integrated analysis of scRNA-seq and scGlycan-seq data, a weighted nearest neighbor (WNN) approach was applied. The FindMultiModalNeighbors() function was used to identify nearest neighbors based on a weighted combination of RNA and lectin features. For WNN analysis, the first 20 principal components from the RNA modality and the first 15 principal components from the lectin modality were used based on elbow-plot inspection. UMAP visualization was generated based on the combined RNA and lectin information using default parameters. Finally, clustering was performed on the WNN graph using the FindClusters() function with a resolution of 1.5.

### Training data

Cell types in the training dataset were annotated based on established marker gene expression from the literature. These included markers for CAFs (*C3*, *C7*, *ACTA2*, *NDUFA4L2*), endothelial cells (*VWF*, *PECAM1*), B cells (*CD79A*), TAMs (*CD86*, *MRC1*, *CD163*), T cells (*RPL21*, *RPL31*, *CD27*, *TIGIT*, *CTLA4*, *CD44*, *CD69*, *GZMK*, *PTPRC*, *SELL*, *CCR7*). Cancer cell subtypes were annotated based on Moffitt marker genes for the “Classical” (*BTNL8*, *FAM3D*, *PRR15L*, *AGR3*, *CTSE*, *LYZ*, *TFF2*, *TFF1*, *ANXA10*, *LGALS4*, *PLA2G10*, *CEACAM6*, *VSIG2*, *TSPAN8*, *ST6GALNAC1*, *AGR2*, *TFF3*, *CYP3A7*, *MYO1A*, *CLRN3*, *KRT20*, *CDH17*, *SPINK4*, *REG4*) and “Basal-like” (*VGLL1*, *UCA1*, *S100A2*, *LY6D*, *SPRR3*, *SPRR1B*, *LEMD1*, *KRT15*, *CTSV*, *DHRS9*, *AREG*, *CST6*, *SERPINB3*, *KRT6A*, *SERPINB4*, *FAM83A*, *SCEL*, *FGFBP1*, *KRT7*, *KRT17*, *GPR87*, *TNS4*, *SLC2A1*, *ANXA8L1*) subtype (Moffitt et al. 2015) and the Raghavan marker genes for the “Intermediate” (*VCAM1*, *MALAT1*, *IGFBP3*, *SYNE2*, *TIMP2*, *TNFAIP2*, *NFAT5*, *SERPINA1*, *CXCL14*, *CX3CL1*, *ALPK3*, *PGGHG*, *DPYSL2*, *TMEM139*, *KLF6*, *KRT23*, *MEGF6*, *HOXB2*, *PADI1*, *MFSD4A*, *INPP4B*, *BAIAP3*, *ARHGAP45*, *ECE1*, *CAMK2N1*, *WFDC2*, *TSHZ2*, *RILPL2*, *LAMB2*) subtype (Raghavan et al. 2021).

### Machine learning

To enable cell-type annotation based solely on glycan features, we developed a supervised machine learning framework using lectin-binding data derived from scGR-seq. A curated panel of 29 lectins capturing diverse glycan-binding specificities was used as input features, while previously annotated cell identities served as reference labels.

Single-cell lectin-binding intensities were extracted from the glycan assay and restricted to a predefined set of lectins. Each cell was represented as a feature vector of normalized lectin-binding signals. Importantly, scaling parameters (mean and standard deviation) were computed exclusively on the training set and subsequently applied to the test set to prevent information leakage.

### Hierarchical classification strategy

We implemented a multi-layer hierarchical classification approach to reflect biological organization and improve prediction accuracy.

At Layer 1, cells were classified as blood or non-blood cells. At Layer 2, blood cells were assigned to the immune-cell branch, whereas non-blood cells were assigned to the cancer- or stromal-cell branch. At Layer 3, cells were classified into one of eight terminal categories: T cells, B cells, TAMs, classical cancer cells, intermediate cancer cells, basal-like cancer cells, CAFs, or endothelial cells.

For all classification layers, we employed gradient boosting decision trees implemented in the XGBoost framework, which is well suited for structured tabular data and can capture non-linear relationships between lectin-binding features and cell identity (Chen and Guestrin 2016).

Model performance was primarily evaluated using leave-one-patient-out validation. In each validation fold, cells from four patients were used for model training, whereas all cells from the remaining patient were reserved as an independent test set. This procedure was repeated five times so that each patient served once as the held-out test patient. All preprocessing parameters, including feature scaling, model fitting, hyperparameter selection, and early stopping, were determined using only the training-patient data in each fold. Predictions from the five held-out test folds were pooled to calculate the overall performance metrics and construct the final confusion matrix. A stratified 80:20 cell-level split was used only as a preliminary analysis and was not used as the principal estimate of model generalizability.

Multi-class classification models were trained using a softmax objective function, with hyperparameters chosen to ensure robust performance (learning rate = 0.1, maximum tree depth = 6, subsampling = 0.8). Early stopping was applied using a validation subset derived exclusively from the training data.

### Layer 1: Major cell type classification

The first model was trained to predict major cell types from lectin-binding profiles. Predictions were assigned based on the maximum class probability, with a confidence threshold of 0.5 applied to label low-confidence predictions as “unclassified.” Model performance was evaluated using classification accuracy and confusion matrices.

### Layers 2 and 3: Subtype classification

To achieve higher-resolution annotation, a second set of classifiers was trained independently for each major cell type. For each group, only cells belonging to that category were used to train a dedicated model to distinguish among its subtypes. In cases where only a single subtype was present, classification was assigned directly without model training.

During inference, each cell was first assigned a major cell type (Layer 1). If the prediction exceeded the confidence threshold, the corresponding subtype classifier (Layer 2 and 3) was applied. Otherwise, the cell was labeled as unclassified. This hierarchical routing strategy improved classification specificity while reducing misclassification across biologically distinct lineages.

A cell was designated as unclassified if the maximum predicted probability was ≤0.5 at any hierarchical decision layer. Such cells were not passed to subsequent classifiers. For calculation of overall eight-class accuracy, unclassified cells were retained in the denominator and counted as incorrect predictions. Coverage was calculated as the number of cells assigned to one of the eight terminal classes divided by the total number of test cells. Confusion matrices were generated to evaluate classification performance across categories, and one-versus-rest ROC analyses were performed to assess discriminative performance for each terminal cell class.

All analyses were performed in R using the Seurat framework for data handling and the XGBoost library for model training. Trained models, including both major and subtype classifiers, were saved for reproducibility and downstream application to independent datasets.

### Biological function of PVL

To generate the input dataset, transcriptomic and lectin-binding matrices were extracted from the Seurat object. Cells were stratified according to their PVL lectin-binding intensity. In the primary analysis, cells within the highest 20% of PVL-binding intensity were designated as PVL-high (label = 1), whereas cells within the lowest 20% were designated as PVL-low (label = 0). These labels were incorporated into the Seurat metadata and used for downstream machine-learning analyses. To evaluate the robustness of the identified PVL-associated transcriptional program, the analysis was repeated using a more stringent definition, in which the top and bottom 10% of cells were classified as PVL-high and PVL-low, respectively.

For the primary analysis, model development, performance evaluation, and feature discovery were conducted using leave-one-patient-out validation. In each validation round, cells from four patients were used for model training, whereas all cells from the remaining patient were held out as an independent test set. This procedure was repeated iteratively so that each patient served once as the held-out test patient. All preprocessing, model training, and feature selection procedures were performed using only the training-patient data within each validation round. Predictions from all held-out test sets were combined for downstream performance evaluation and biological interpretation. The previously performed 70:30 cell-level split was retained only as a preliminary analysis and was not used as the primary assessment of model generalizability. All analyses were performed in R using the Seurat, nnet, caret, iml, and xgboost packages.

To identify transcriptional features associated with PVL-binding status, we employed a single-hidden-layer neural network. Because the primary objective of this analysis was biological feature discovery rather than classifier optimization or benchmarking of machine-learning algorithms, we intentionally adopted a simple and interpretable neural-network architecture. Specifically, a multilayer perceptron (MLP) model implemented in the nnet package was trained using normalized gene-expression values as input features. The model consisted of a single hidden layer with three neurons and an output layer configured for binary classification of PVL-high and PVL-low cells using a softmax activation function. This framework was selected to facilitate biological interpretation of PVL-associated transcriptional programs through subsequent SHAP (Shapley Additive Explanations) analysis.

Model training was performed with a maximum of 500 iterations and L2 regularization (weight decay = 1 × 10^−4^). Classification performance was evaluated on independent test datasets using confusion matrices generated with the caret package.

To assess the contribution of individual genes to PVL classification, SHAP (Shapley Additive Explanations) analysis was performed using the iml package. SHAP values were calculated for individual cells and averaged across PVL-high cells to identify genes contributing most strongly to PVL-high classification. The highest-ranking SHAP genes were exported for downstream survival analysis and functional interpretation, including pathway enrichment analyses.

### Correlation analysis between PVL binding and gene expression

To assess the continuous relationship between PVL-binding intensity and transcriptional features, Spearman’s rank correlation coefficients were calculated between normalized PVL-binding intensity and the expression levels of the top 20 SHAP-prioritized genes across PDAC cancer cells. PVL-binding intensity was treated as a continuous variable. Statistical significance was evaluated using two-sided tests, and significant correlations were visualized as a heatmap (Supplementary Fig. 10).

### Pathway enrichment analysis

To investigate the biological functions associated with PVL-binding status, pathway enrichment analyses were performed using the top 200 SHAP-prioritized genes identified from the neural-network model. Over-representation analysis was conducted using the Hallmark gene sets from the Molecular Signatures Database (MSigDB) and the Kyoto Encyclopedia of Genes and Genomes (KEGG) pathway database. Enrichment significance was evaluated using a hypergeometric test, and P values were adjusted for multiple testing using the Benjamini–Hochberg method. Pathways with an adjusted P value <0.05 were considered significantly enriched.

### Survival analysis

To evaluate the clinical relevance of the PVL-associated transcriptional program, survival analysis was performed using the TCGA pancreatic adenocarcinoma (PAAD) cohort. The signature score was calculated as the mean z-score of the 200 SHAP-prioritized genes. Patients were stratified into high- and low-signature groups based on the median signature score. Overall survival was evaluated using Kaplan–Meier analysis, and statistical significance between groups was assessed using the log-rank test.

## Supplementary Material

Supplementary_materials_cwag100

## Data Availability

All model parameters, trained models, and analysis scripts are available from the corresponding author upon reasonable request. The processed lectin-binding matrix, de-identified patient identifiers, cell-type annotations, model parameters, trained models, and analysis scripts are available through Zenodo record 22,976,393. The deposited files and metadata required to reproduce the principal analyses are described in the accompanying repository documentation.
