# Supplementary material for: scGlycoAI: machine learning analysis of single-cell glycan and RNA sequencing data for cell-type annotation and malignancy-associated glycan discovery
Source: Glycobiology. 2026 Oct 24;36(11):cwag100. doi: 10.1093/glycob/cwag100 (PMC13718437; doi:10.1093/glycob/cwag100)
Supplement: Supplementary file 1 [file supplementary_materials_cwag100.zip › 260922-Supplemmentary information.pdf]

Supporting Information

scGlycoAI: Machine learning analysis of single-cell glycan and RNA sequencing data for cell-type annotation and malignancy-associated glycan discovery

Supplementary Figure S1-S10

Dinh Xuan Tuan Anh, Hiroaki Tateno

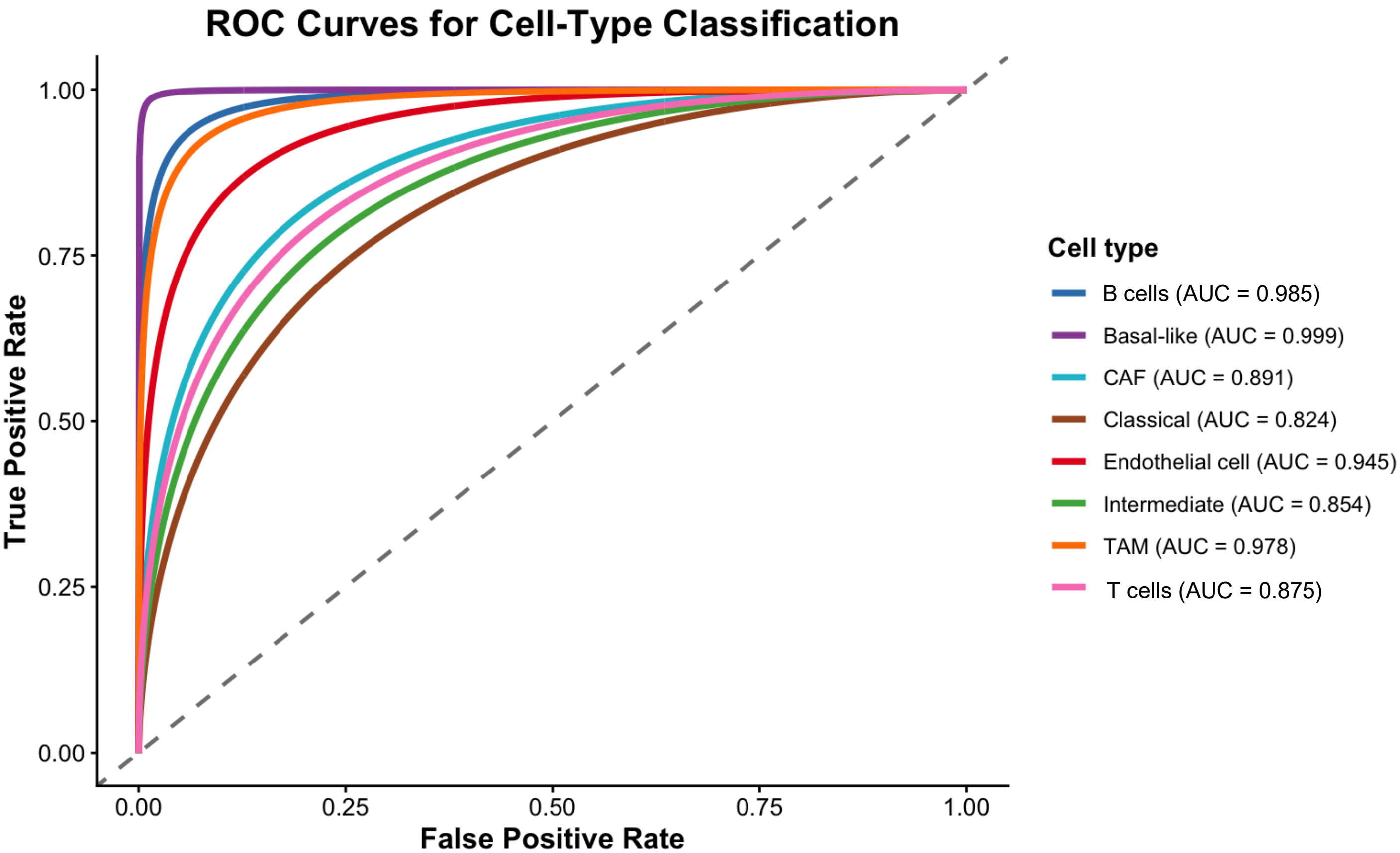

Figure S1. Lectin-based machine learning enables cell-type classification. Receiver operating characteristic (ROC) curves showing the performance of a machine-learning classifier using lectin-binding profiles to distinguish individual cell types. ROC curves were generated using a one-versus-rest strategy, with the area under the ROC curve (AUC) indicated for each cell type. Higher AUC values indicate stronger discrimination between the indicated cell type and all other cell populations.

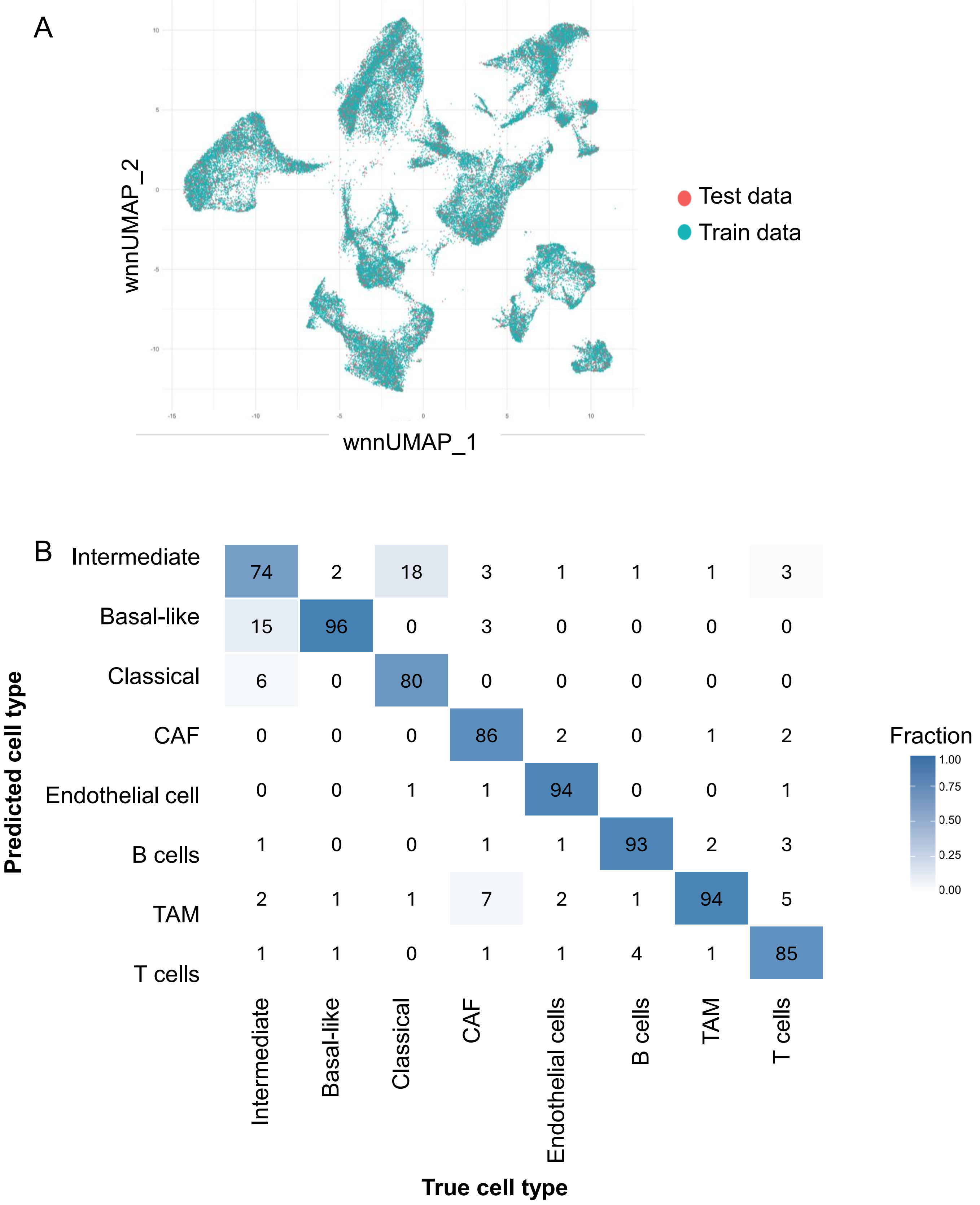

Figure S2. Evaluation of scGlycoAI annotation. (A) UMAP visualization of the training and testing datasets used for scGlycoAI annotation, with cells from training (80%) and test (20%) . (B) Confusion matrix for Layer 1 annotation, showing classification performance across major cell types.

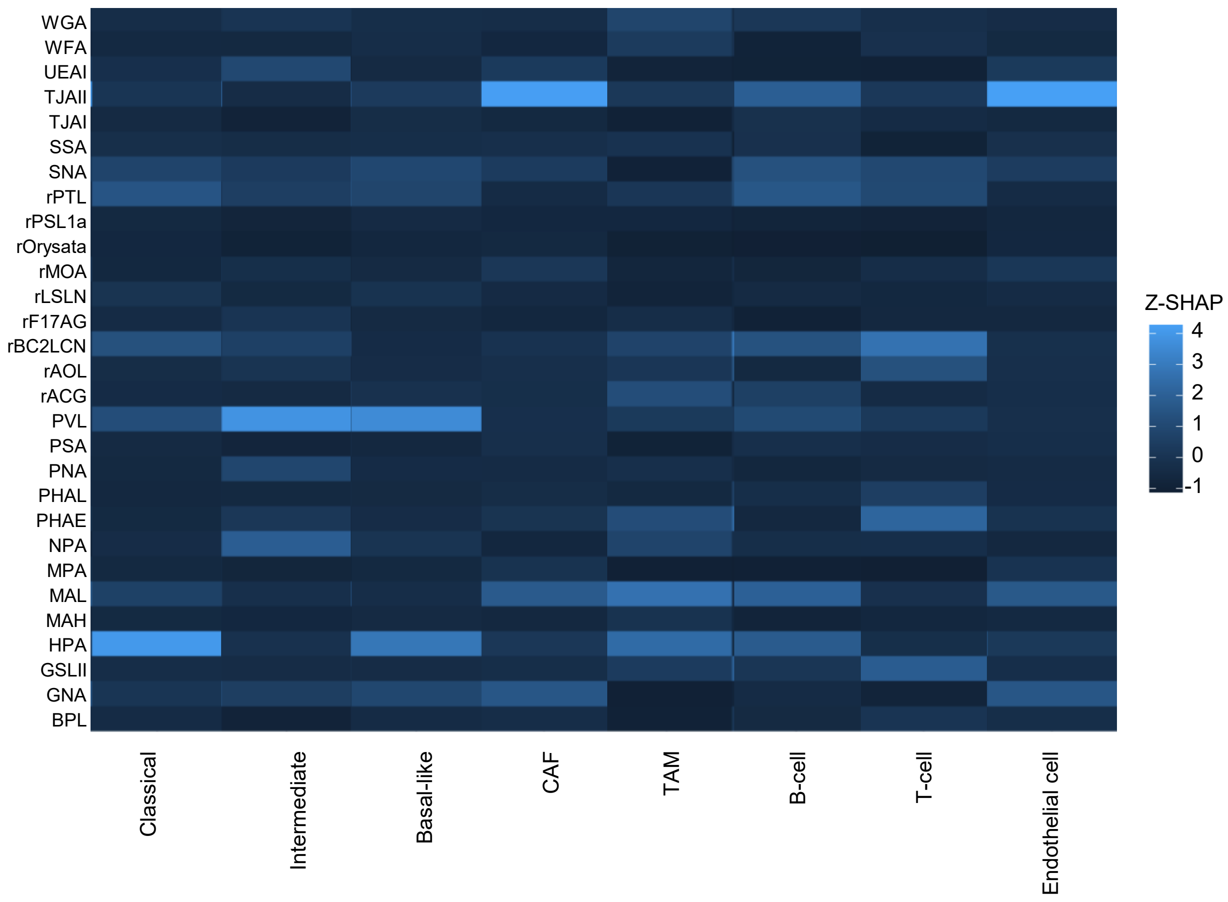

Figure S3. Heatmap showing the relative contributions and importance of individual lectins for cell type classification and annotation.

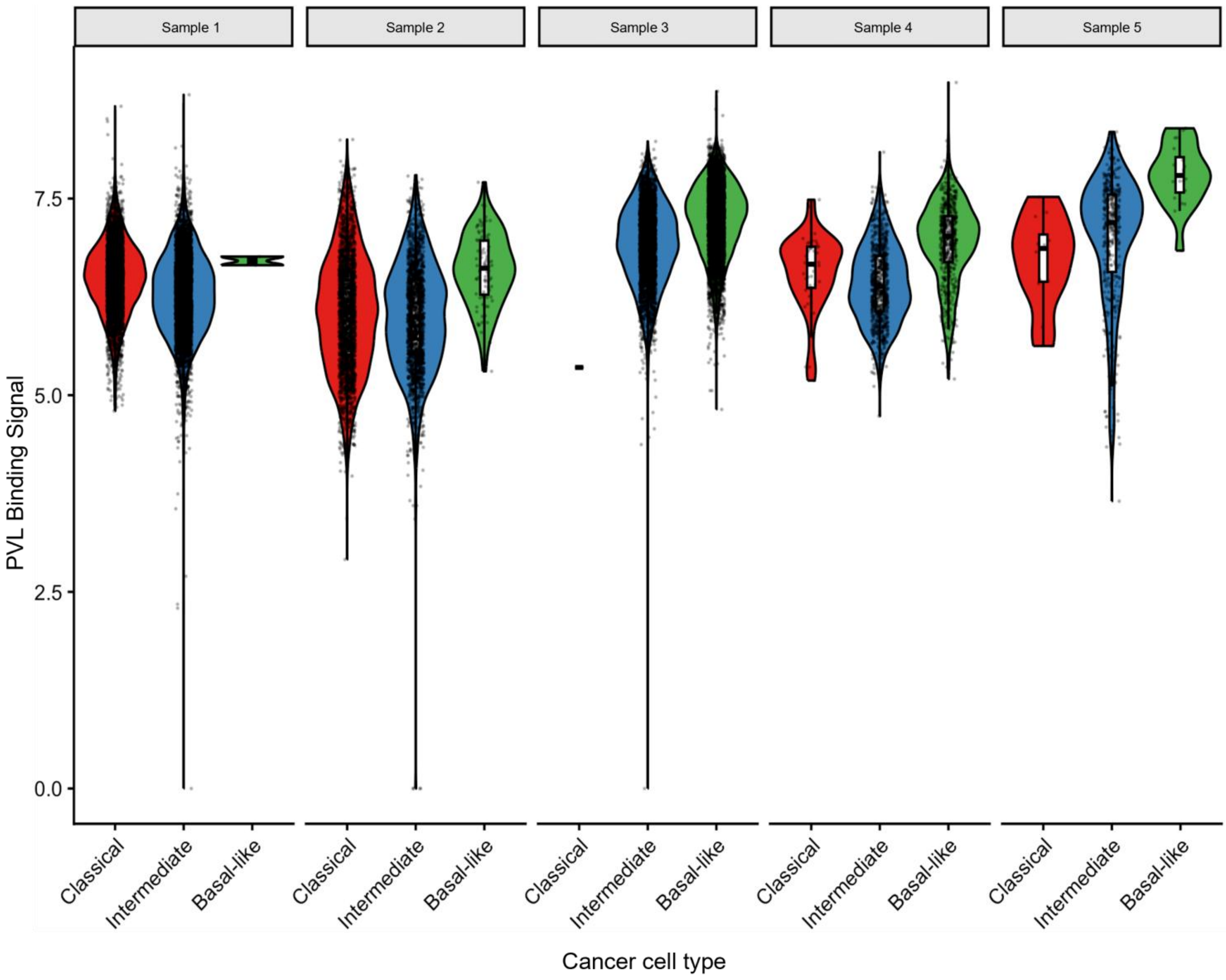

Figure S4. Violin plots showing single-cell PVL-binding intensity across pancreatic cancer-cell subtypes within each patient. Superimposed points indicate patient-level summary values [specify mean or median].

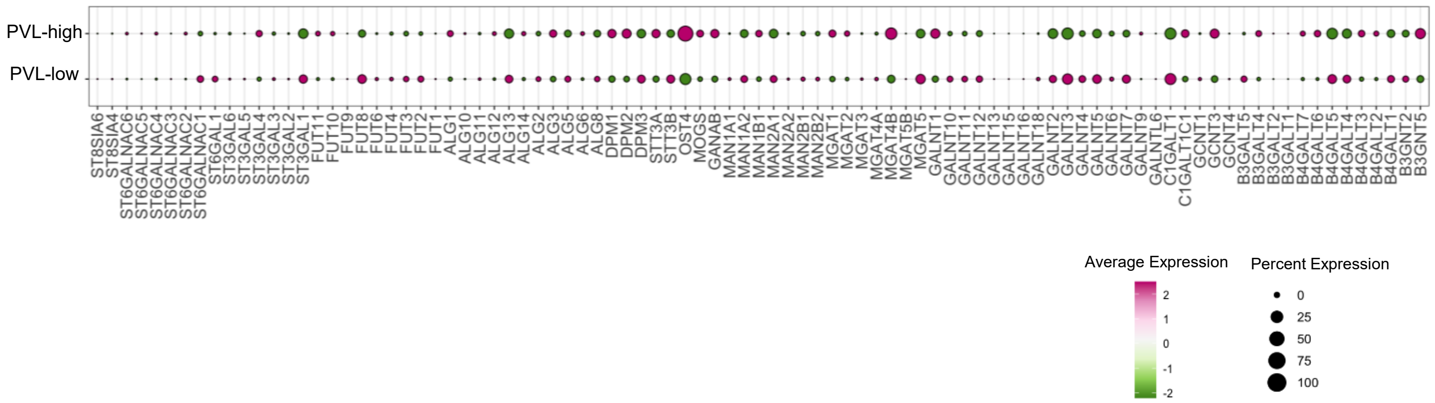

Figure S5. Dot plots of the average expression of glycosyltransferase genes for PVL-high and PVL-low obtained from scGR-seq data. The x-axis represents glycosyltransferase genes. The y-axis represents cell types. “Percentage Expressed” indicates the percentage of single cells in each cell type expressing the gene detected ranging from 0 to 100. “Average Expression” indicates the average expression level of the genes in each cell type.

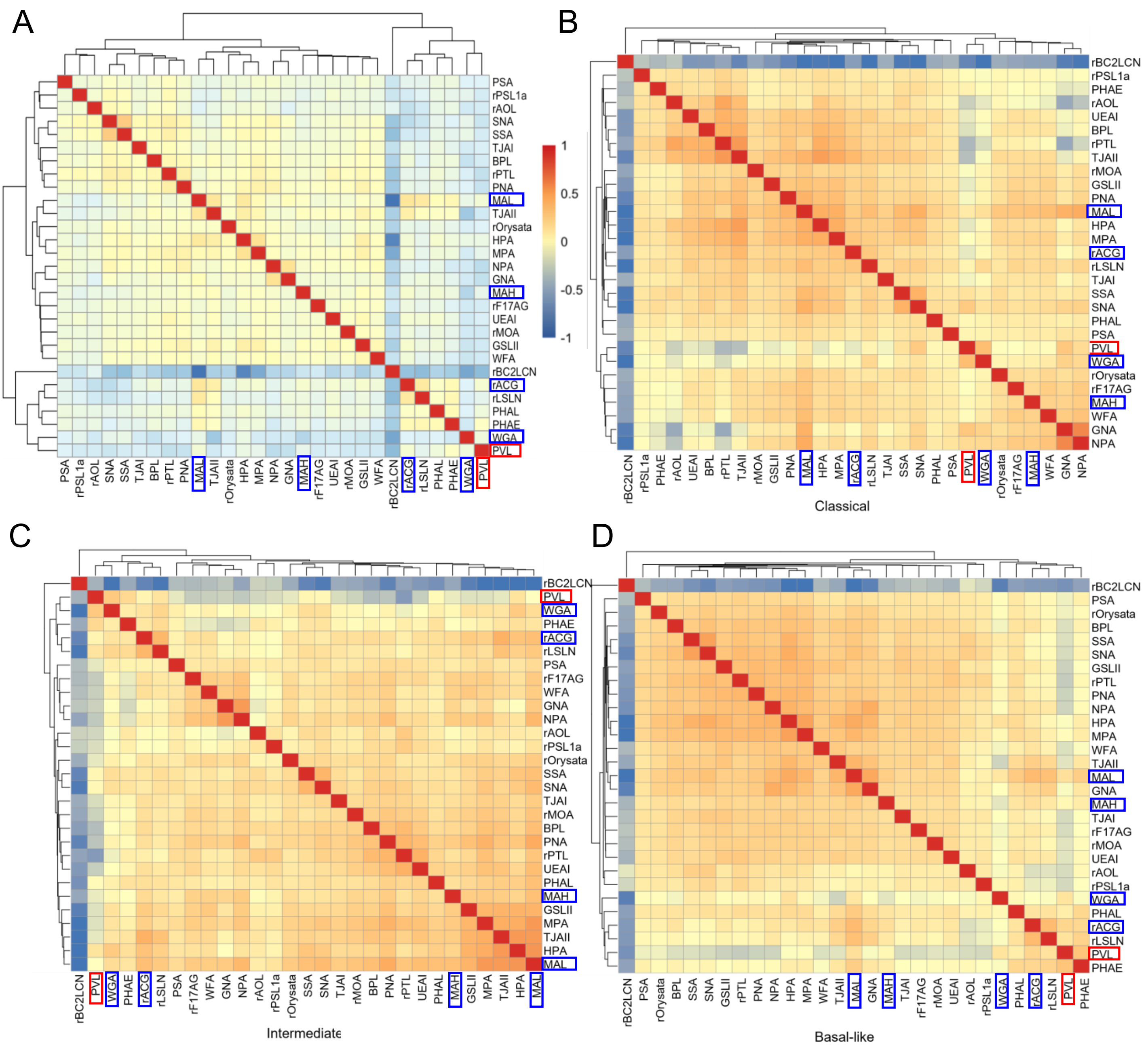

Figure S6. Heatmaps showing Spearman correlation coefficients among lectin-binding signals within (A) PVL-high cells, (B) classical, (C) intermediate, and (D) basal-like cancer cells. The color scale represents Spearman's ρ.

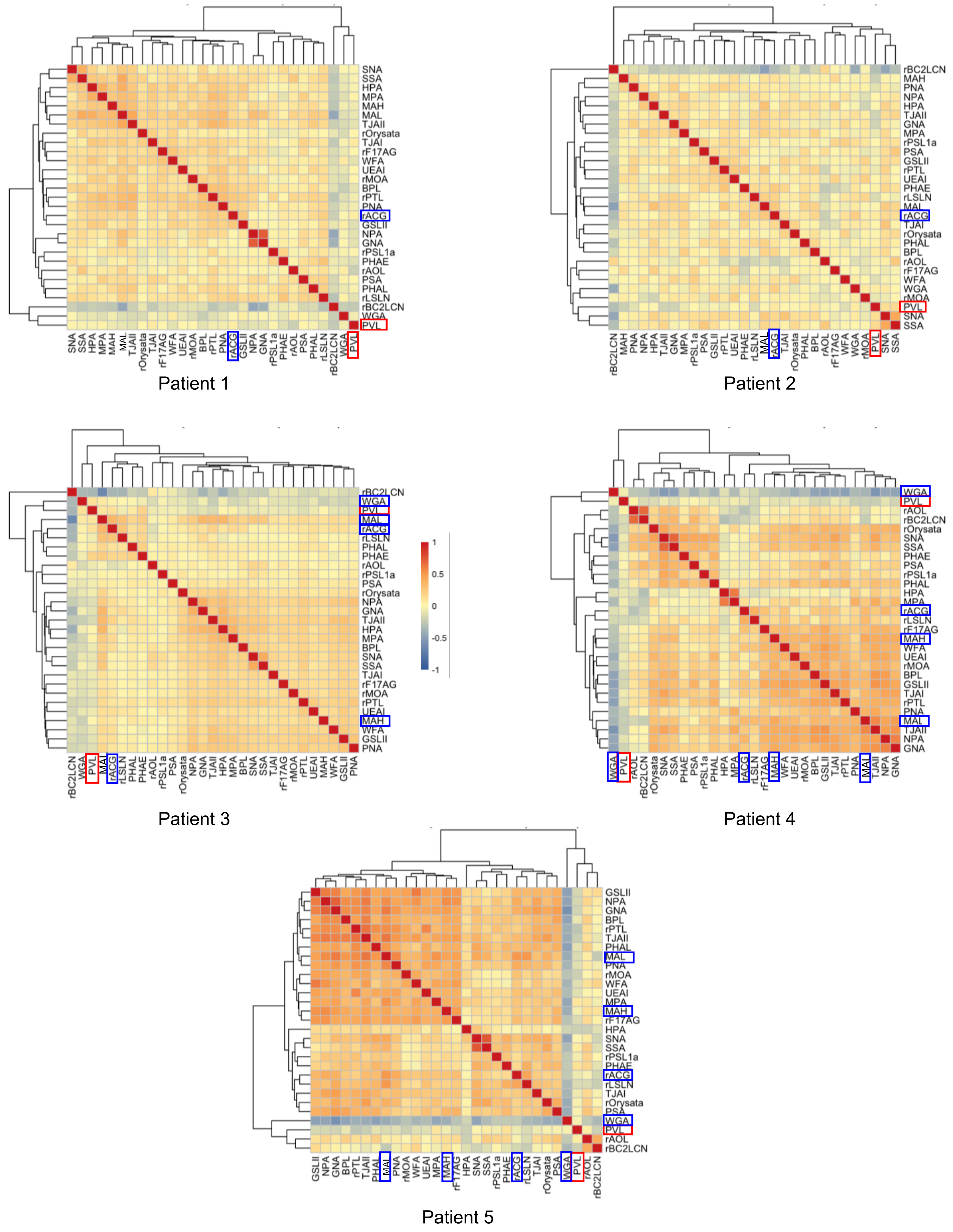

Figure S7. Heatmaps showing Spearman correlation coefficients among lectin-binding signals across individual patients. Color intensity represents the magnitude and direction of Spearman's correlation coefficient (ρ).

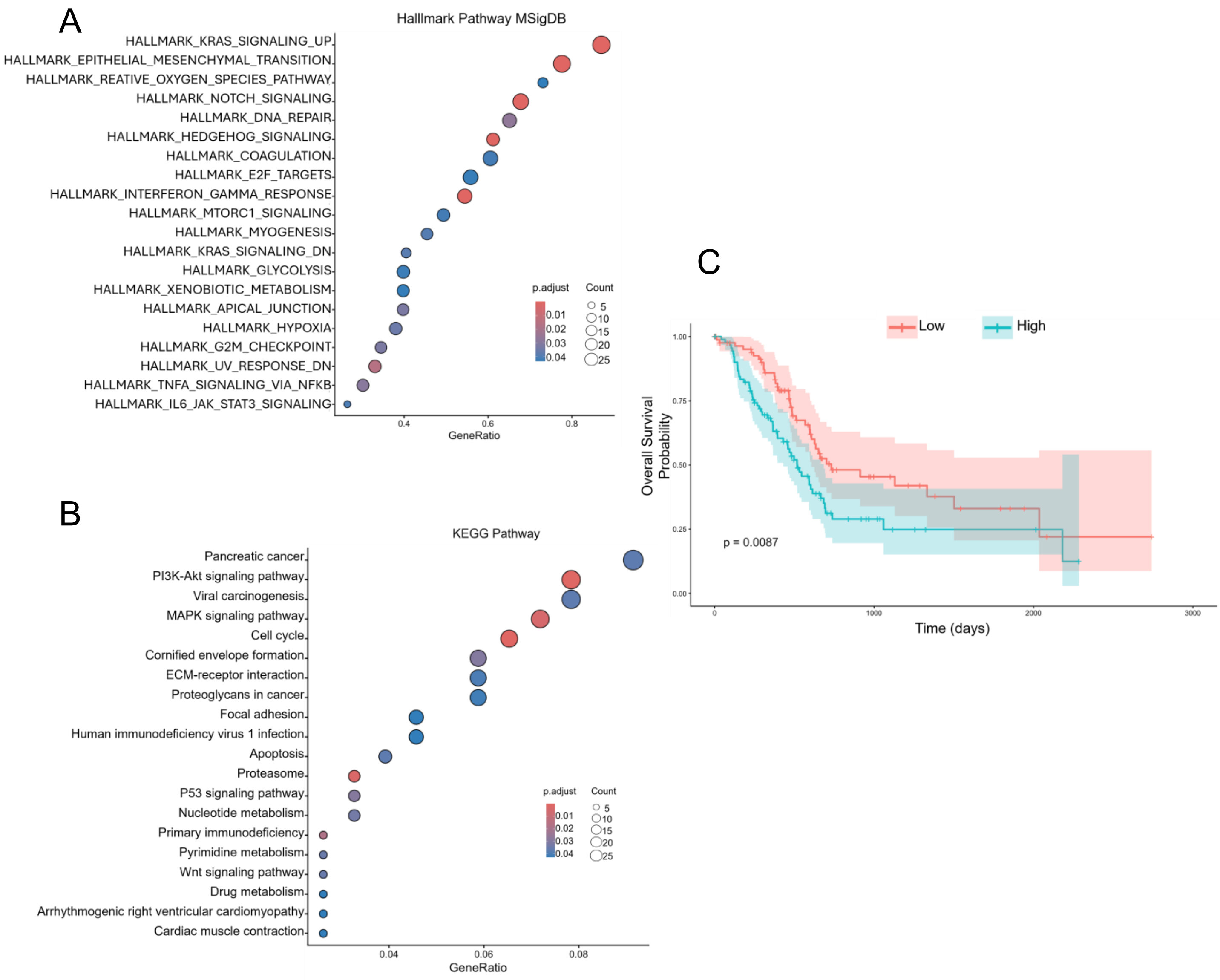

Figure S8. (A, B) Hallmark and KEGG pathway enrichment analyses of the top 200 SHAP-prioritized genes associated with the PVL-high group. (C) Kaplan–Meier overall survival analysis of pancreatic adenocarcinoma (PAAD) patients stratified according to the expression levels of the 200 SHAP-associated genes identified in the PVL-high group. PVL-high cells were defined based on the top 10% of PVL lectin-binding intensity, and the predictive model was evaluated using patient-level data splitting to ensure independent training and validation.

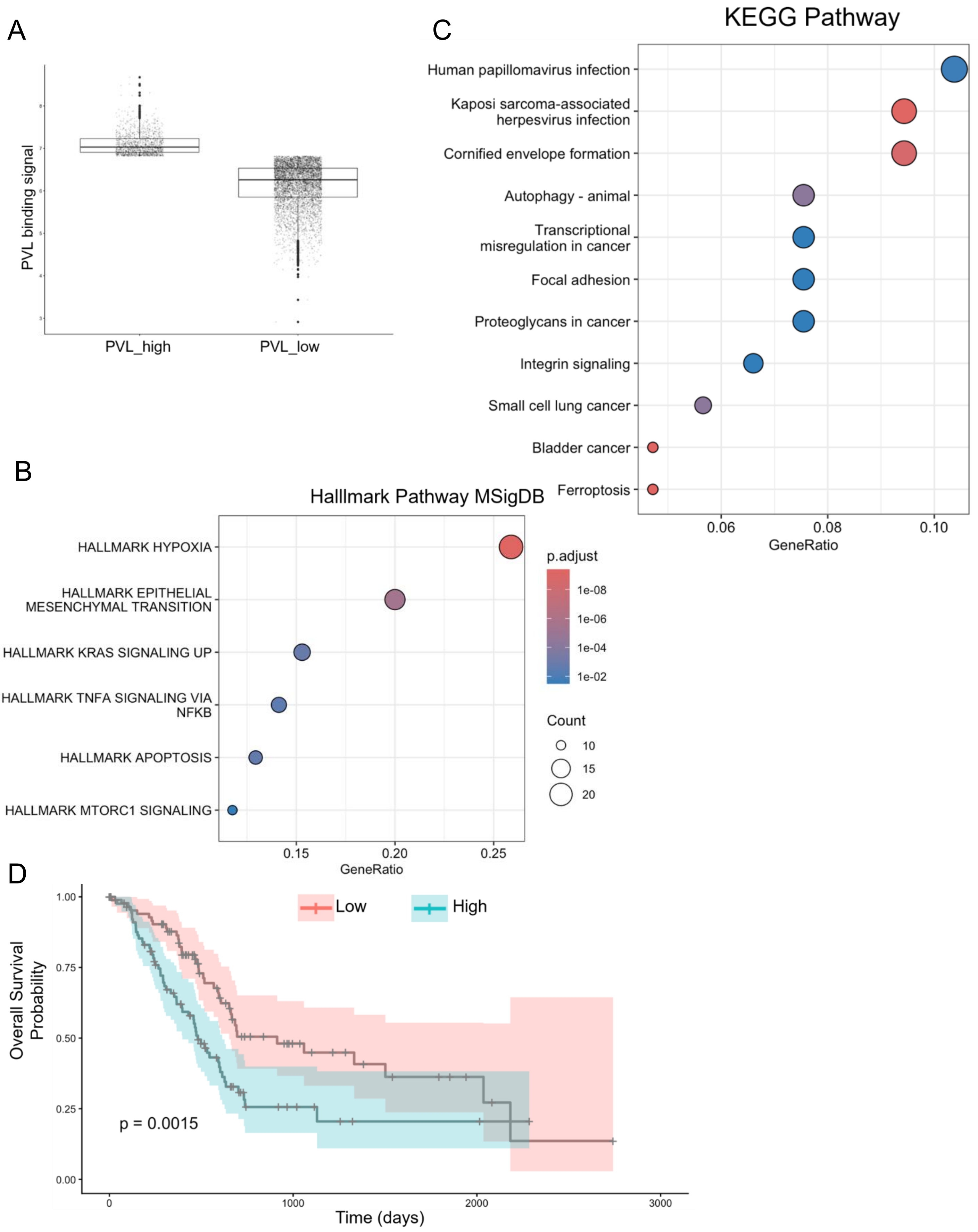

Figure S9. (A) PVL groups in classical cancer cells. (B, C) Hallmark and KEGG pathway enrichment analyses of the top 200 SHAP-prioritized genes associated with the PVL-high group. (D) Kaplan–Meier overall survival analysis of pancreatic adenocarcinoma (PDAC) patients

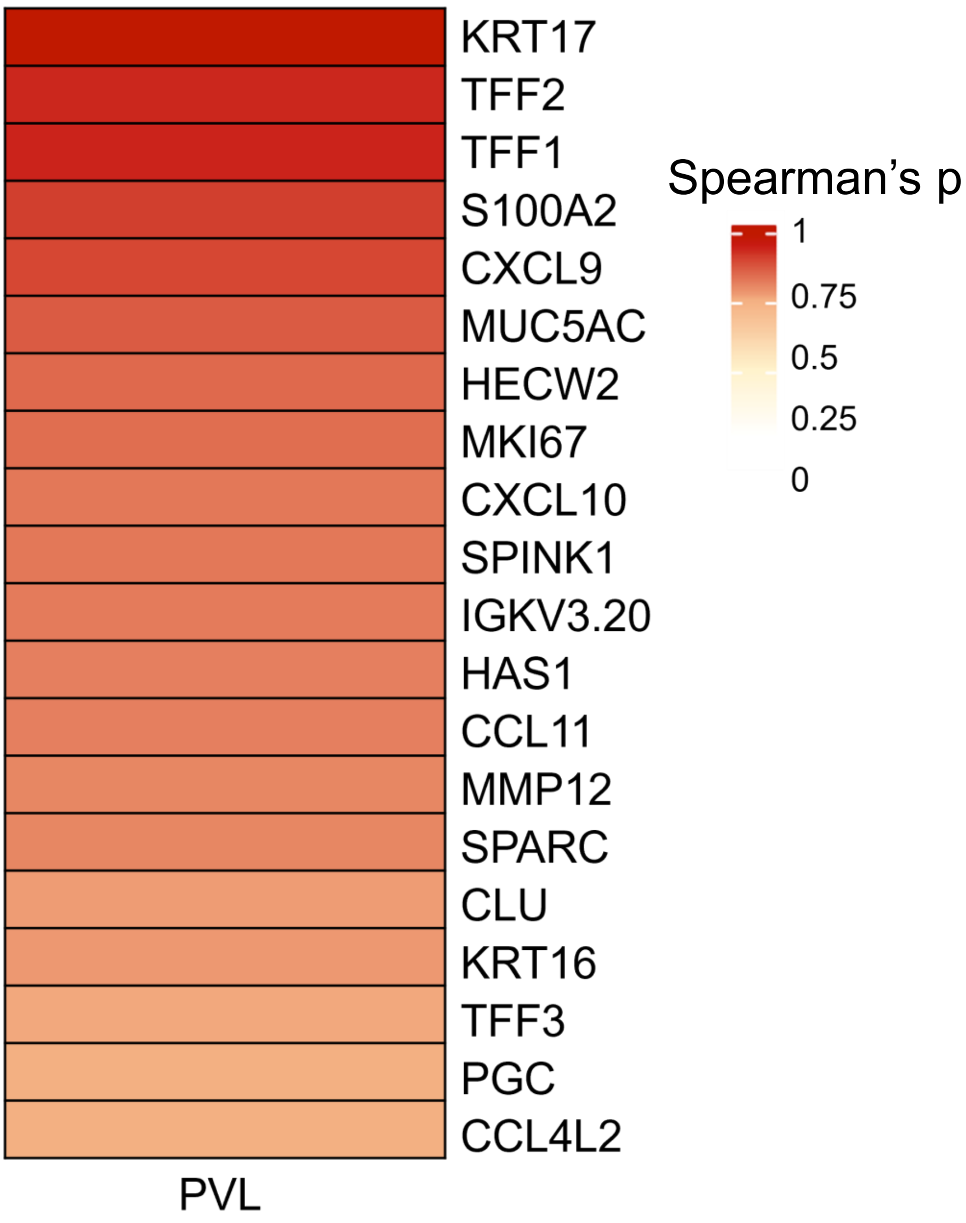

Figure S10. Heatmap showing Spearman's rank correlation coefficients (ρ) between PVL-binding intensity and the normalized expression levels of the top 20 SHAP-prioritized genes across PDAC cancer cells. PVL-binding intensity was analyzed as a continuous variable. The color scale represents Spearman's ρ, where positive values indicate that higher gene expression is associated with higher PVL-binding intensity and negative values indicate an inverse association. The observed coefficients ranged from 0.74 to 0.98. Statistical significance and adjusted P values are provided in Supplementary Table 2.

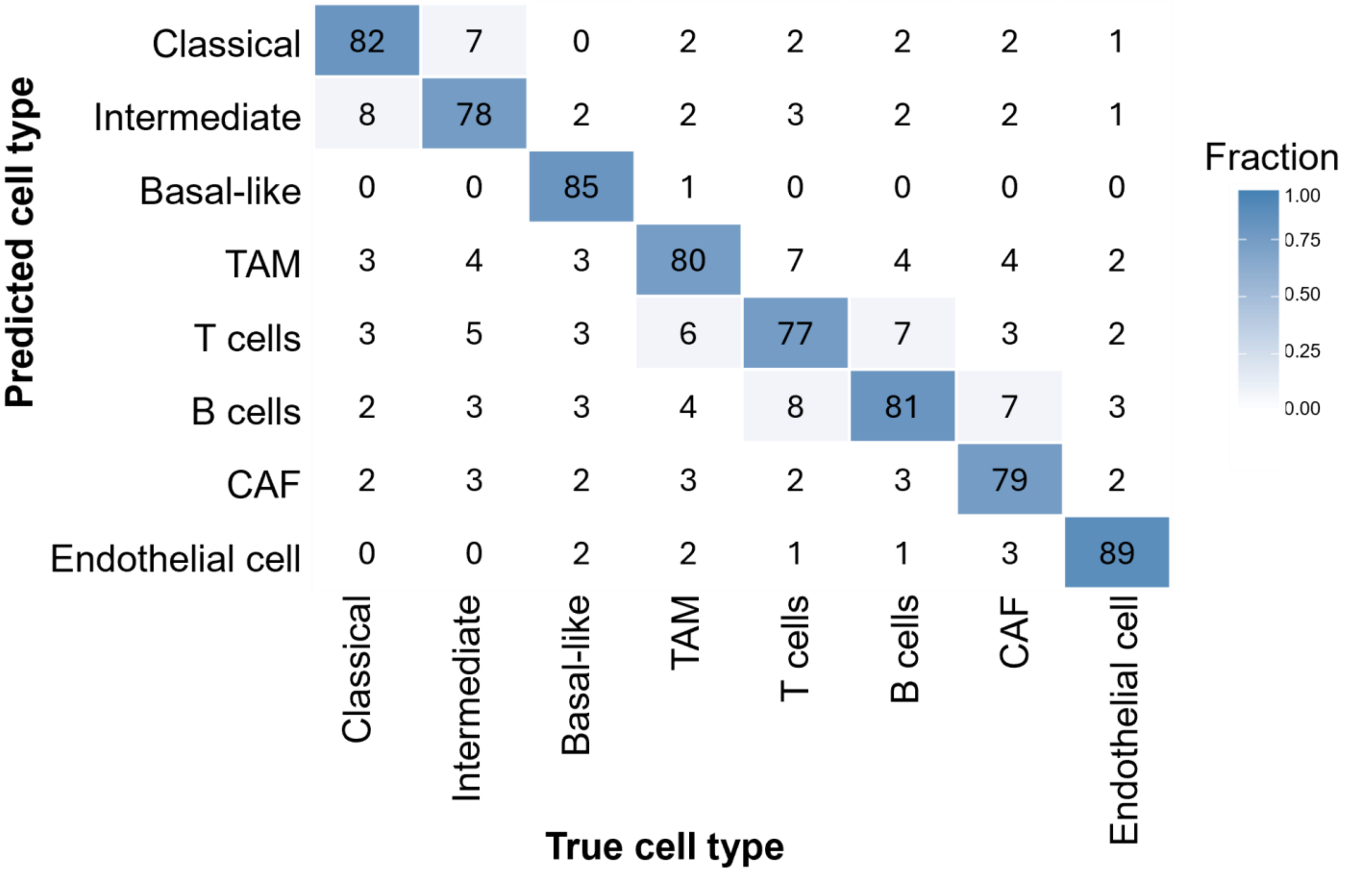

Figure S11. Patient-level evaluation of scGlycoAI annotation performance, showing the confusion matrix for the conventional multiclass model.
